# Ultraviolet Imaging with Low Cost Smartphone Sensors: Development and Application of a Raspberry Pi-Based UV Camera

**DOI:** 10.3390/s16101649

**Published:** 2016-10-06

**Authors:** Thomas C. Wilkes, Andrew J. S. McGonigle, Tom D. Pering, Angus J. Taggart, Benjamin S. White, Robert G. Bryant, Jon R. Willmott

**Affiliations:** 1 Department of Geography, The University of Sheffield, Winter Street, Sheffield S10 2TN, UK; a.mcgonigle@sheffield.ac.uk (A.J.S.M.); t.pering@sheffield.ac.uk (T.D.P.); a.taggart@sheffield.ac.uk (A.J.T.); r.g.bryant@sheffield.ac.uk (R.G.B.); 2 Istituto Nazionale di Geofisica e Vulcanologia, Sezione di Palermo, via Ugo La Malfa 153, Palermo 90146, Italy; 3 Department of Electronic and Electrical Engineering, The University of Sheffield, Portobello Centre, Pitt Street, Sheffield S1 4ET, UK; ben.white@sheffield.ac.uk (B.S.W.); j.r.willmott@sheffield.ac.uk (J.R.W.)

**Keywords:** UV camera, UV imaging, smartphone sensor technology, sulphur dioxide emissions, Raspberry Pi, low-cost camera

## Abstract

Here, we report, for what we believe to be the first time, on the modification of a low cost sensor, designed for the smartphone camera market, to develop an ultraviolet (UV) camera system. This was achieved via adaptation of Raspberry Pi cameras, which are based on back-illuminated complementary metal-oxide semiconductor (CMOS) sensors, and we demonstrated the utility of these devices for applications at wavelengths as low as 310 nm, by remotely sensing power station smokestack emissions in this spectral region. Given the very low cost of these units, ≈ USD 25, they are suitable for widespread proliferation in a variety of UV imaging applications, e.g., in atmospheric science, volcanology, forensics and surface smoothness measurements.

## 1. Introduction

Ultraviolet (UV) imaging has a wide variety of scientific, industrial and medical applications, for instance in forensics [1], industrial fault inspection [2], astronomy, monitoring skin conditions [3] and in remote sensing [4,5]. To date, scientific grade UV cameras, which have elevated quantum efficiencies in this spectral region, have been applied in this context. However, these systems are relatively expensive (typical unit costs thousands of dollars) and can be power intensive, since they may incorporate thermo-electric cooling. Although these units may provide high signal-to-noise ratios, a lower price point solution could expedite more widespread implementation of UV imaging.

Recently, considerable effort has been invested in developing low cost back-illuminated complementary metal-oxide semiconductor (CMOS) sensor technology. Previously, this sensor architecture was applied only within specialist, low light imaging arenas, e.g., in astronomy. In the last few years, however, the manufacturing costs of these devices have been reduced markedly, such that they now feature prominently in consumer electronic products, particularly smartphones. The key advantage of the back-illuminated sensor architecture, over the conventional CMOS configuration, is that the photo-diodes are placed in front of the metal wiring matrix layer of the sensor, thereby improving fill factor and substantially increasing optical throughput to the photoreceptors, particularly in the UV, where these detectors are photosensitive.

To date, however, application of these inexpensive sensors has predominantly been focused on visible imaging, due to the choice of fore-optics and the Bayer filter layer applied to the sensors, in order to generate red-green-blue (RGB) mosaics. Recent studies have highlighted that these smartphone cameras do have some UV sensitivity [6,7,8], even with the above optical arrangement. Here, we build on this work by modifying such a camera sensor, to maximize UV throughput to the sensor. To the best of our knowledge, this constitutes the first report of an imaging system specifically adapted for the UV, based on a back-illuminated CMOS device developed for the smartphone market. In particular, we developed a UV camera system, based on Raspberry Pi camera boards, and demonstrated its utility for UV imaging applications down to 310 nm, via a case study involving remote sensing of sulphur dioxide (SO_2_) emissions from a power station smokestack.

## 2. UV Camera Development

This work was achieved using Raspberry Pi Camera Module v1.3 boards (referred to as PiCams hereafter), of cost ≈ USD 25 (Raspberry Pi Foundation). The PiCam is based on an Omnivision OV5647 back-illuminated CMOS sensor, developed primarily for the mobile phone market; the OV5647 is a 1/4” 5-megapixel (2592 × 1944 active array) backlit-CMOS unit, with 8-/10-bit RGB/RAW image output. PiCam boards were chosen here due to the ease of data acquisition and image processing using Raspberry Pi computer boards (Raspberry Pi Foundation), via the Python programming language. Due to their low expense and power consumption, Raspberry Pi computers are increasingly being used in a variety of sensing applications (e.g., [9,10]). Whilst this work is focused on one sensor type, preliminary tests with another such back-illuminated CMOS device, the Sony IMX219 (Sony Corporation, Minato, Tokyo, Japan), also evidence UV sensitivity.

**Figure 1:**
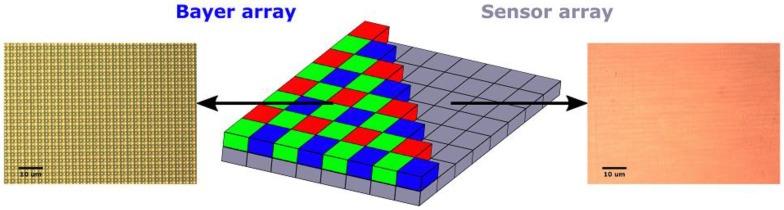
A schematic (**Centre**) of the Bayer filter array and its positioning on the photodetector array. Also shown are microscope images of the PiCam sensor pre- (**Left**) and post- (**Right**) Bayer removal process.

The PiCam lens and filter, housed above the sensor within a plastic casing unit, both absorb the UV signal, and were therefore removed as a first step to developing the UV camera. This was achieved chemically using a Posistrip**^®^** EKC830^TM^ (DuPont, Wilmington, DE, USA) bath. To improve UV sensitivity of the sensor, we then removed the microlens and Bayer filter layers, the latter of which masks the sensor in a mosaic of RGB colour filters (Figure 1), and attenuates much of the incident UV radiation. This process effectively turns the PiCam into a monochrome sensor.

Whilst the filter can be removed by careful scratching (a range of tools, such as metal tweezers or a pointed wooden object, can be used for this process), a far more uniform finish is achievable chemically; we adopted the latter approach using a five step procedure. We first submerged the sensors in photoresist remover (Posistrip**^®^** EKC830^TM^) and heated (70–100 °C) until the filter was entirely removed; this generally takes 10–30 minutes, depending on the level of applied heating and agitation. A second bath of this remover was then applied to optimise the cleaning procedure. Following this, the photoresist remover was washed from the sensor by a three stage cleaning procedure, using successive baths of n-butyl acetate, acetone and isopropyl alcohol. All the above chemicals were applied undiluted. For each of these steps, the sensor was submerged on the order of minutes, to ensure a thorough cleaning. Careful and rigorous application of this methodology can result in uniform and clean sensors which exhibit greatly enhanced UV sensitivity relative to non-de-Bayered units. For example, a clear-sky image taken with a partially de-Bayered unit, captured through a 310 nm filter and with a shutter speed of 400 ms, exhibits an increase of ≈ 600% in a de-Bayered region relative to the unmodified section of the sensor.

For image capture, it is necessary to retrieve RAW sensor data, rather than the standard JPEG image output, as the former excludes the de-Bayering algorithm and image processing, which results in a non-linear response from the sensor. This, and all the below acquisition and processing steps were achieved with in-house authored Python codes. Here the RAW images were saved to the camera, where they were stored in the metadata of the 8-bit JPEG image. These binary data are then extracted and saved as PNG images, to preserve the 10-bit RAW digital number (DN) format, in files of ≈ 6 MB. These images could then be straightforwardly processed and analysed.

**Figure 2:**
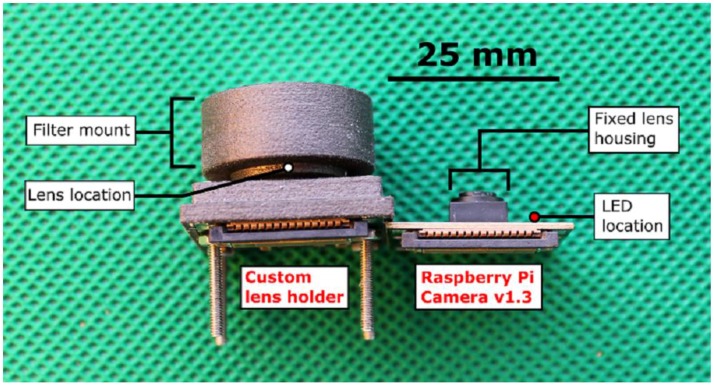
A profile image of the Raspberry Pi Camera Module (**Right**), and the modified system with custom built optics (**Left**). The custom design is bolted to the camera board using the pre-existing mount holes.

A UV transmissive anti-reflection (AR) coated plano-convex quartz lens of 6 mm diameter and 9 mm focal length (Edmund Optics Ltd., Barrington, NJ, USA; $100), was then mounted to the fore of the PiCam, using an in-house designed, 3D printed lens holder, attached to the board using its pre-existing mount holes; this provided a field of view of ≈ 28°. This lens housing consists of two parts, enabling straightforward focusing via screw thread adjustment. As the housing covers the entire camera board, it was necessary to disable the board’s light-emitting diode (LED), which, by default, is programmed to turn on during image acquisition (see Figure 2). By way of comparison: the all in cost of this camera configuration (e.g., PiCam, lens, filter, filter holder, chemical removal costs) including a UV bandpass filter is ≈ USD 200–300, depending on the filter, in comparison to a typical figure of at least ten times the upper value, for such a system based on currently available scientific grade UV cameras.

Following construction of the imaging system, the sensor linearity, which is important for quantitative applications, and UV sensitivity were tested. This was achieved by mounting a UV bandpass filter, of 12.5 mm diameter, centred on 310 nm and of 10 nm full width at half maximum (FWHM) (Edmund Optics Ltd.) to the fore of the camera lens; these filters have no other transmission features in the spectral sensitivity range of the CMOS detectors.

**Figure 3:**
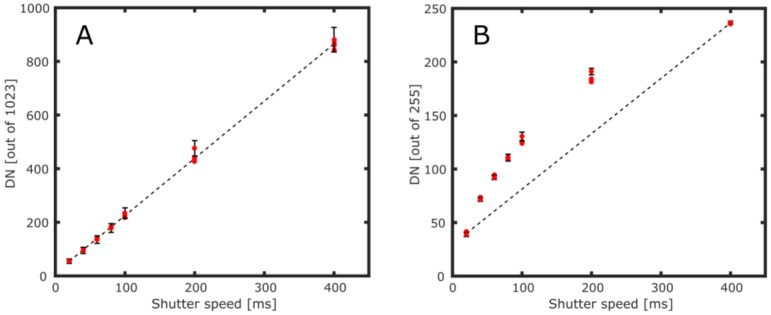
Plots of average pixel signal (in digital number; DN) vs. shutter speed (ms) for a cropped region (800 × 600 pixels) of clear-sky images taken at 310 nm: (**A**) the 10-bit RAW image output shows a linear increase in DN with respect to shutter speed; (**B**) the 8-bit standard output JPEG image shows a non-linear response in all three of the red-green-blue (RGB) channels (Red-channel DNs are plotted here) in line with gamma correction. An error bar is inserted on one data point per exposure time, indicating the standard deviation of the pixel intensities in the cropped region; the bar heights are approximately the same for all points of equivalent shutter speed, and just one bar is displayed for clarity.

To test the UV sensor response, images of uniformly illuminated clear-sky were taken through the 310 nm filter at varying shutter speeds; these experiments were performed in Sheffield, UK, at approximately 11:00 local time (solar zenith angle of around 50°), during February 2016. At each shutter speed four images were captured and analog gain was fixed throughout, for consistency; the operating software does not allow gain specification, but it does enable this to be fixed, following stabilization of the system after the camera start-up process. By averaging pixel DNs from a 800 × 600 pixel region in the centre of each image, we observed a linear increase in average pixel DN relative to shutter speed, for the RAW 10-bit sensor data (Figure 3A). Furthermore, the RAW images demonstrated near saturation at shutter speeds well below 1 s, in a spectral region where there is very little skylight due to ozone absorption, indicating usable UV sensor sensitivity.

## 3. Measurements of Power Station Sulphur Dioxide Emissions

Sulphur dioxide has strong UV absorption bands between 300 and 320 nm [11], which have been exploited in a range of atmospheric remote sensing measurements of this species, using differential optical absorption spectroscopy and UV imagery [12,13,14]. In particular, power stations release SO_2_ to the atmosphere from their smokestacks, and remotely sensing these emissions is one means of ensuring regulatory compliance and constraining effects on the atmosphere [4,5,15,16].

In order to demonstrate proof of concept of the utility of the developed sensors for UV imaging applications, we deployed two UV PiCam units at Drax power station in the United Kingdom over a ≈ 15 min period on 15 August 2016. Bandpass filters, centred at 310 and 330 nm (10 nm FWHM; Edmund Optics Ltd.), were mounted to the fore of the co-aligned cameras, one on one unit, with the other filter on the other camera. Both devices simultaneously imaged the rising smokestack plume. Shutter speeds of ≈ 300 and 40 ms, respectively, were applied for the cameras, in view of the greater scattered skylight intensity at the latter wavelength, and images were acquired at 0.25 Hz. Camera-to-camera pixel mapping was achieved subsequently in software, using the smokestack as a reference. The acquisition and retrieval protocols followed those of Kantzas et al. [17], in particular by collecting dark images for each camera, which were subtracted from all acquired sky images. Clear sky images, taken adjacent to the plume, were also acquired, then normalised to generate a mask, which all acquired plume images were divided by; this eliminates vignetting as well as any sensor non-uniformity which may have resulted from the de-Bayering process. As SO_2_ absorbs at 310 nm, but not at 330 nm, contrasting resulting images provides a means of constraining the spatial distribution of gas concentration across the field of view, whilst eliminating sources of extinction common to both wavelengths, e.g., due to aerosols. In particular, SO_2_ apparent absorbance (*AA*) for each pixel was determined using the following relation [17]:

(1) $$AA=-\log_{10}\left[\frac{IP_{310}}{IB_{310}}/\frac{IP_{330}}{IB_{330}}\right]$$

where *IP* is the in-plume intensity, and *IB* is the background intensity, taken as the average value in the clear sky adjacent to the rising gas plume; the subscripts specify the camera filter in question, for the dark subtracted, mask corrected imagery. Calibration of *AA* was then carried out using quartz cells containing known column amounts of SO_2_ (in our case 0, 300 and 900 ppm.m), in particular measuring *AA* values for each cell when pointing at plume free sky, plotting concentration vs. these, and then multiplying all acquired plume image *AA* values by this gradient. The cell column amounts were verified using an Ocean Optics Inc. USB2000 spectrometer, using the VolcanoSO2 differential optical absorption spectroscopy code [18].

**Figure 4:**
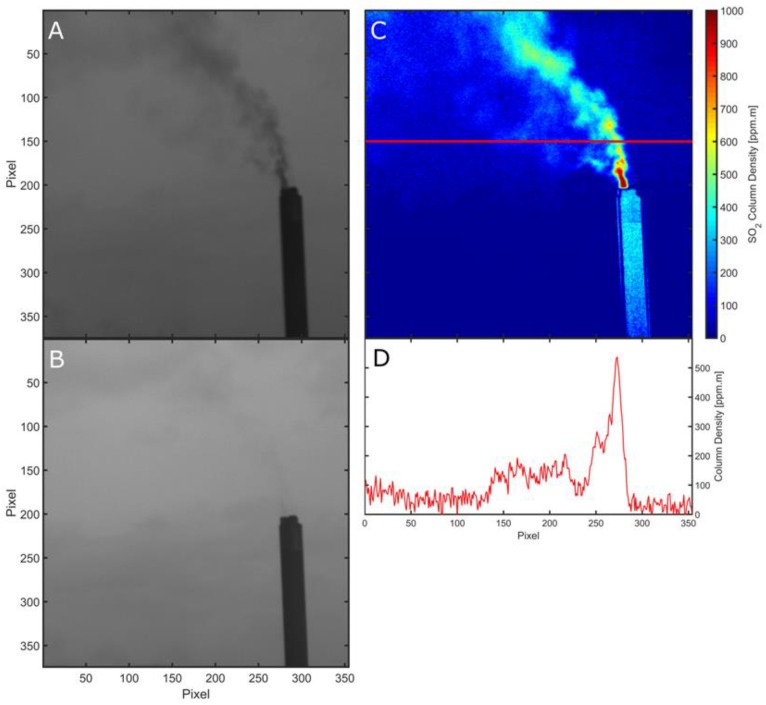
(**A**) A cropped image of the Drax smokestack taken at 310 nm with a shutter speed of 300 ms. The initial image pixels are binned to generate a pixel resolution of 648 × 486, to reduce noise; dark image subtraction and mask corrections have been applied. (**B**) As in (A) but at 330 nm with a shutter speed of 40 ms. (**C**) The resulting calibrated SO_2_ image of Drax power station stack and plume showing the clear capacity of the system to resolve the plume emissions. (**D**) A cross-section of (C) showing gas concentrations along the row delineated by the red line. The background noise level can be clearly observed between pixels 300 to 350.

Figure 4C shows a typical calibrated SO2 image, after binning pixels to a 648 × 486 array to reduce noise, highlighting the ability of the cameras to clearly resolve the smokestack emissions. Furthermore, background image noise levels are remarkably low (typically ≈ 25 ppm.m standard deviation in a 648 × 486 binned image), in contrast to values of ≈ 30 ppm.m, previously reported from more expensive scientific grade camera systems in atmospheric SO_2_ monitoring [5], indicating the potential of these low cost units in UV imaging applications.

**Figure 5:**
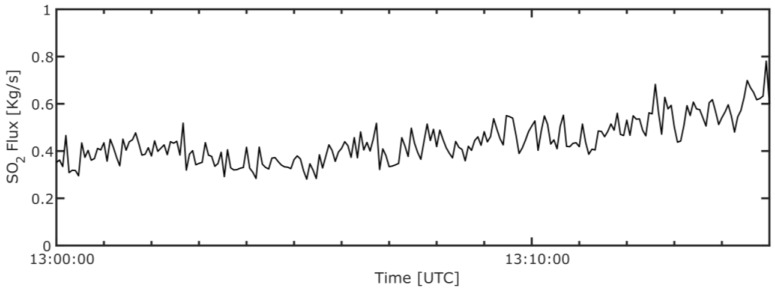
Time series of SO_2_ flux from Drax power station for a 15 min acquisition period.

By integrating across the plume, perpendicular to the plume transport direction, integrated column amounts (ICAs) of SO_2_ were obtained, and a time series of ICAs through successive images was produced. A plume speed of ≈4.9 m/s was calculated by generating two such ICA time series, determined for parallel plume cross sections, located at different distances from the source, and then cross correlating these. This plume speed was then multiplied by the ICA values to generate a SO_2_ flux time series, as shown in Figure 5. The SO_2_ flux was found to be relatively stable from the smokestack, with a mean emission of 0.44 kg/s; nevertheless, notable gas “puffs” are apparent, and can be seen both in the flux time series and SO_2_ absorption image video (see Video S1 in the supplementary materials). The Drax Annual Reviews of Environmental Performance [19], provide the best available ancillary emissions data for comparison, stating annual SO_2_ loadings ranging 24.5–35.1 kt/yr for the period 2008–2013, and thus, average annual fluxes ranging 0.78–1.1 kg/s. These data corroborate our observations, falling within an order of magnitude, given that Drax’s operational output will fall somewhat below the mean annual value during the British summer.

## 4. Discussion and Concluding Remarks

In this paper we have presented, for the first time, a relatively simple methodology for the development of low-cost UV cameras, based on inexpensive sensors developed for the smartphone market. We show that by modification of Raspberry Pi camera sensors, and rebuilding of the optical systems, camera boards as cheap as ≈ USD 25 can be adapted to applications down to wavelengths of at least 310 nm. The potential utility of such devices in UV imaging applications was illustrated via a case study, in which we used the cameras to perform ultraviolet remote sensing of SO_2_ fluxes from a power station smokestack.

With this in mind, other possible areas in which this technology could be applied include, but are not limited to: ground-based mineral aerosol detection (331/360 nm), to mimic satellite based TOMS retrievals [20,21], monitoring skin conditions [3,22], forensics during crime scene investigation [1,23], fault detection in power systems [2], fault detection in vehicles, and astronomy. The low cost would be of particular benefit in application areas where budgets are limited, or arrays of these units are required. One example of such a scenario is in the field of volcanology, where UV cameras are used to image SO_2_ emissions in volcanic plumes [4,24,25] as a means of investigating magmatic processes. As many volcanoes are located in developing countries, lower cost sensors could greatly expedite more comprehensive monitoring of global volcanic hazards, with allowance for sensor destruction in the event of explosions.

One potential current limitation to this system is its maximum achievable frame rate. The shutter speeds used here at the power station (300 ms at 310 nm) correspond to a theoretically achievable acquisition rate of >1 Hz. However, in reality obtainable frame rates are rather lower due to on chip processing. Preliminary tests suggest that framerates >0.5 Hz are achievable, however, over prolonged periods this resulted in sporadic dropping of frames, which disappeared altogether for acquisition at 0.25 Hz. More work is required to identify the limiting factor in frame capture, and whether stable higher acquisition rates might be achievable with further software optimisation. For the majority of applications, however, we suggest that framerates of 0.25 Hz will be more than adequate.

Future work could focus on the spectral range of such units; the sensitivity of this system below 310 nm has not been quantified herein, and whilst the sensors do likely respond at deeper UV wavelengths, there may be a trade off in terms of signal to noise. Investigating the longer wavelength cut off of these monochrome devices could also be of interest, in establishing their use in high temperature thermal imaging applications. Other work may include consideration of thermal effects on sensor stability/noise, and the degree to which cooling/temperature stabilisation might improve signal to noise. Finally, whilst signal to noise levels appear promising here, particularly given the low sensor price point, further work is now also merited in comparing the system sensitivity and noise characteristics against more expensive scientific grade UV cameras.

## Supplementary Materials

The following are available online at http://www.mdpi.com/1424-8220/16/10/1649/s1, Video S1: Absorbance image sequence of Drax SO_2_ emissions. Warmer colours indicate larger SO_2_ column densities. Images were captured at 0.25 Hz, and this video sequence is speeded up to eight times real-time.
